# Supplementary material for: 4D analysis of malaria parasite invasion offers insights into erythrocyte membrane remodeling and parasitophorous vacuole formation
Source: Nat Commun. 2021 Jun 15;12:3620. doi: 10.1038/s41467-021-23626-7 (PMC8206130; doi:10.1038/s41467-021-23626-7)
Supplement: Supplementary file 1 — Supplementary Information [file 41467_2021_23626_MOESM1_ESM.pdf]

Supplementary Information

4D analysis of malaria parasite invasion offers insights into erythrocyte membrane remodeling and parasitophorous vacuole formation

Niall D. Geoghegan[1,2,5], Cindy Evelyn[1,2,5], Lachlan W. Whitehead[1,2], Michal Pasternak[1,2,3,4], Phoebe McDonald[1,2], Tony Triglia[1,2], Danushka S. Marapana[1,2], Daryan Kempe[4], Jennifer K. Thompson[1], Michael J. Mlodzianoski[1,2], Julie Healer[1,2], Maté Biro[4], Alan F. Cowman[1,2], Kelly L. Rogers[1,2*]

[1]The Walter & Eliza Hall Institute of Medical Research, Parkville, VIC, Australia.

[2]Department of Medical Biology, The University of Melbourne, Parkville, VIC, Australia.

[3]Imperial College London, London SW7 2AZ, UK.

[4]EMBL Australia, Single Molecule Science node, School of Medical Sciences, University of New South Wales, Sydney, Australia.

[5]These authors contributed equally: Niall D. Geoghegan, Cindy Evelyn

[*] Correspondence: rogers@wehi.edu.au

Supplementary Figures

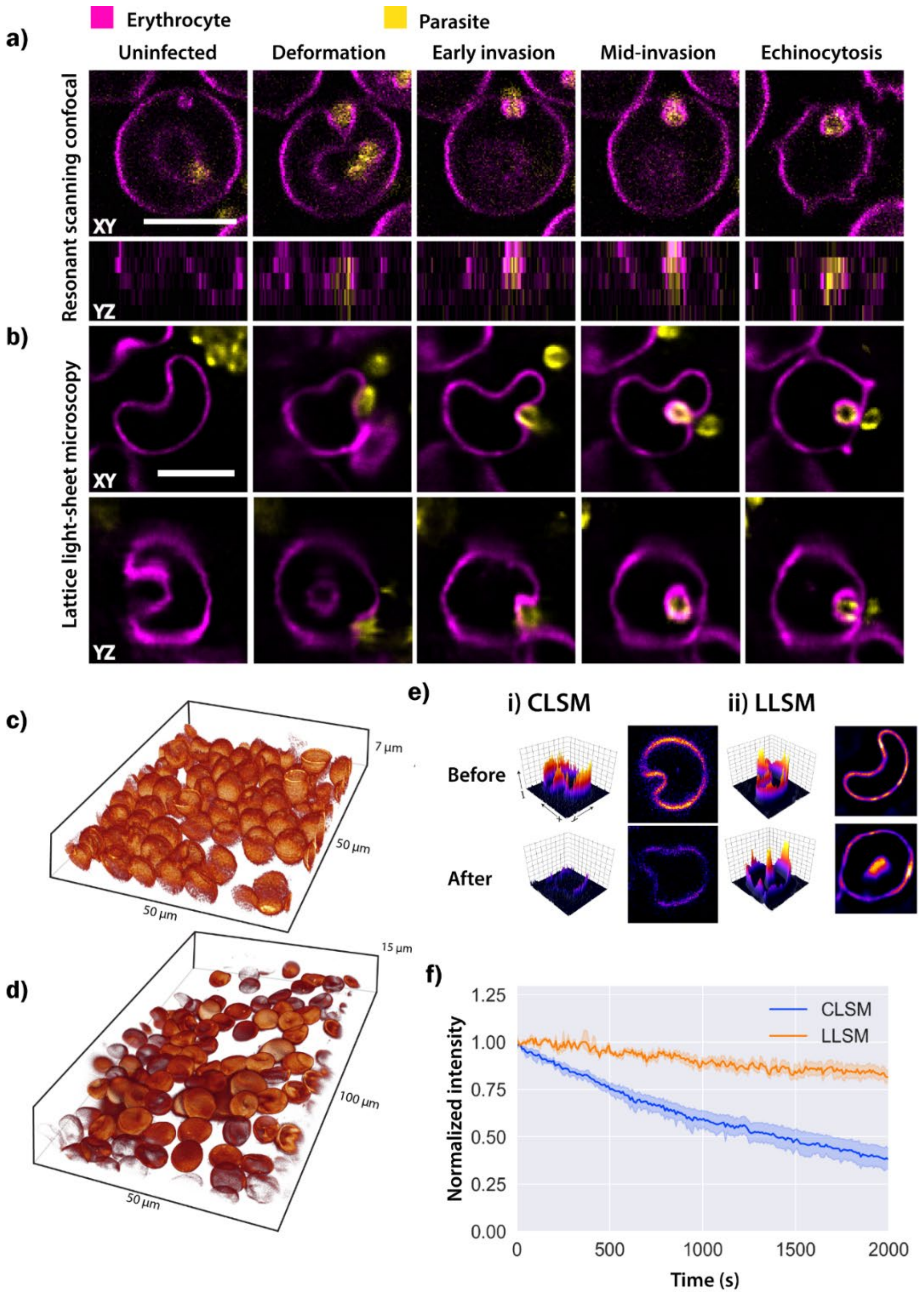

Supplementary Figure 1. Lattice light-sheet microscopy versus confocal laser scanning microscopy. Cross-sectional views showing the XY and YZ coordinates of data generated by **a)** the resonant scanning confocal (representative of 8 independent experiments) and **b)** the lattice

light-sheet microscope (representative of 9 independent experiments) during different stages of invasion. Images represent parasites labelled with MitoTracker Red or Mitotracker Deep Red, yellow, interacting with erythrocytes labelled with Di-4-ANEPPDHQ, magenta. Scale bars: 6 µm. 3D volume projections of **c)** confocal laser scanning microscopy (CLSM) versus **d)** lattice light-sheet microscopy (LLSM). **e)** 3D (x,y,I) plots plus corresponding 2D images, with fire look-up table, before and after scanning the cell for a duration of 2000 s, showing the relative SNR for **(e(i))** confocal and **(e(ii))** lattice light-sheet microscopy data. **f)** Plots showing the normalized intensity value for confocal (blue line) versus lattice light sheet (orange line) over 2000 s, from which the data at **e(i)** and **e(ii)** are derived. Data presented as mean (line) ± 95% Confidence Interval (shaded region).

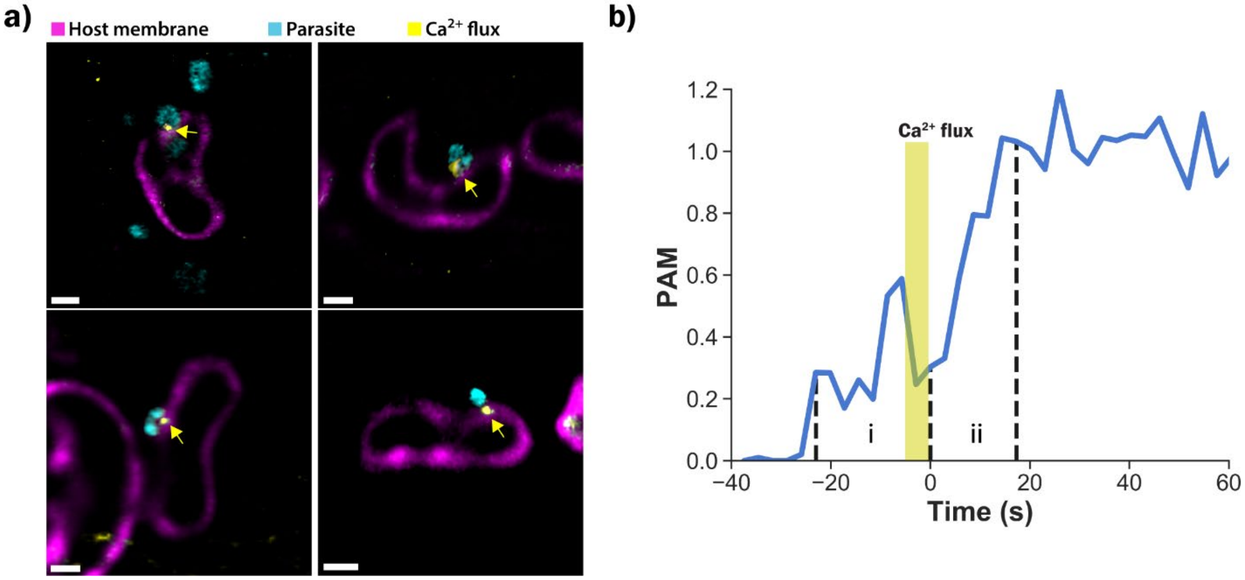

Supplementary Figure 2. Observation of the Ca^{2+} flux during invasion using lattice light sheet microscopy. a) 2D cross sections of the punctate Ca^{2+} flux show the localization of the flux at the parasite apical tip. Views represent the best orientation of a cross section for 4 different invasion events from three independent experiments. Images represent parasites labelled with MitoTracker Red or MitoTracker Deep Red, cyan, interacting with erythrocytes labelled with Di-4-ANEPPDHQ, magenta, and loaded with Fluo-4AM Ca^{2+} reporter, yellow. Scale bars: 2 µm. **b)** The parasite associated membrane (PAM) time-plot (n=11 from three independent experiments) shows a conserved timing of the Ca^{2+} flux (shown by the yellow bar) which marks the transition from deformation (i) to internalization (ii).

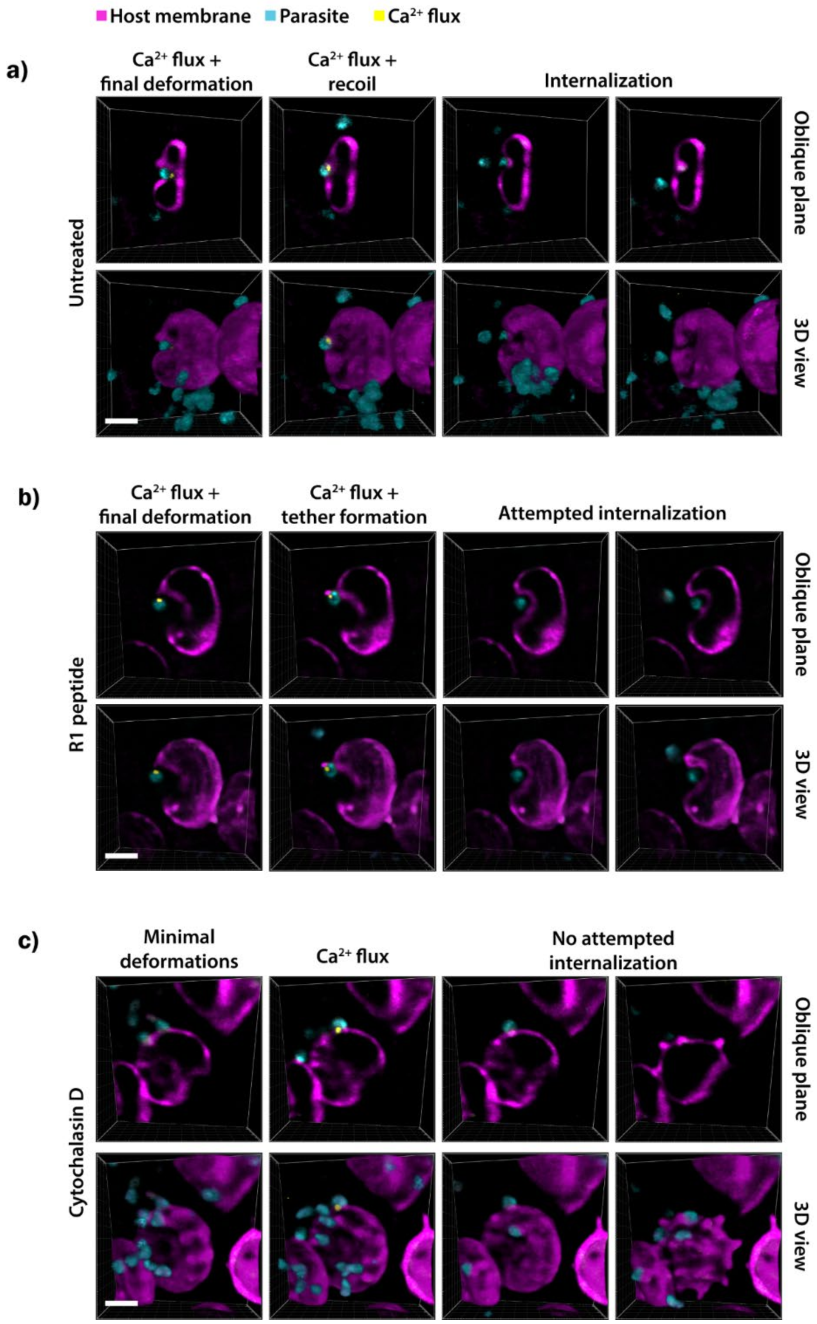

Supplementary Figure 3. Ca^{2+} flux serves as a temporal marker to study inhibition effects that occur downstream from PfRh5-basigin interaction. Images represent parasites labelled with MitoTracker Red or MitoTracker Deep Red, cyan, interacting with erythrocytes labelled with Di-4-ANEPPDHQ, magenta, and loaded with Fluo-4AM Ca^{2+} reporter, yellow. The experiment

was repeated two times for cytochalasin D treatment and three times for each untreated and R1 peptide treatment. Snapshots displayed in blend mode with oblique plane or 3D view using IMARIS. **a)** Ca^{2+} flux occurred during a final deformation and recoil of the erythrocyte membrane, followed by a successful internalization. **b)** In the presence of R1 peptide, Ca^{2+} flux coincided with a tether formation from the erythrocyte membrane at the parasite-erythrocyte interaction site. The parasite deformed the erythrocyte strongly after the Ca^{2+} flux, without successful internalization. **c)** In the presence of cytochalasin D, the parasite did not cause any observable deformation before and after Ca^{2+} flux. Scale bars: 3 μm.

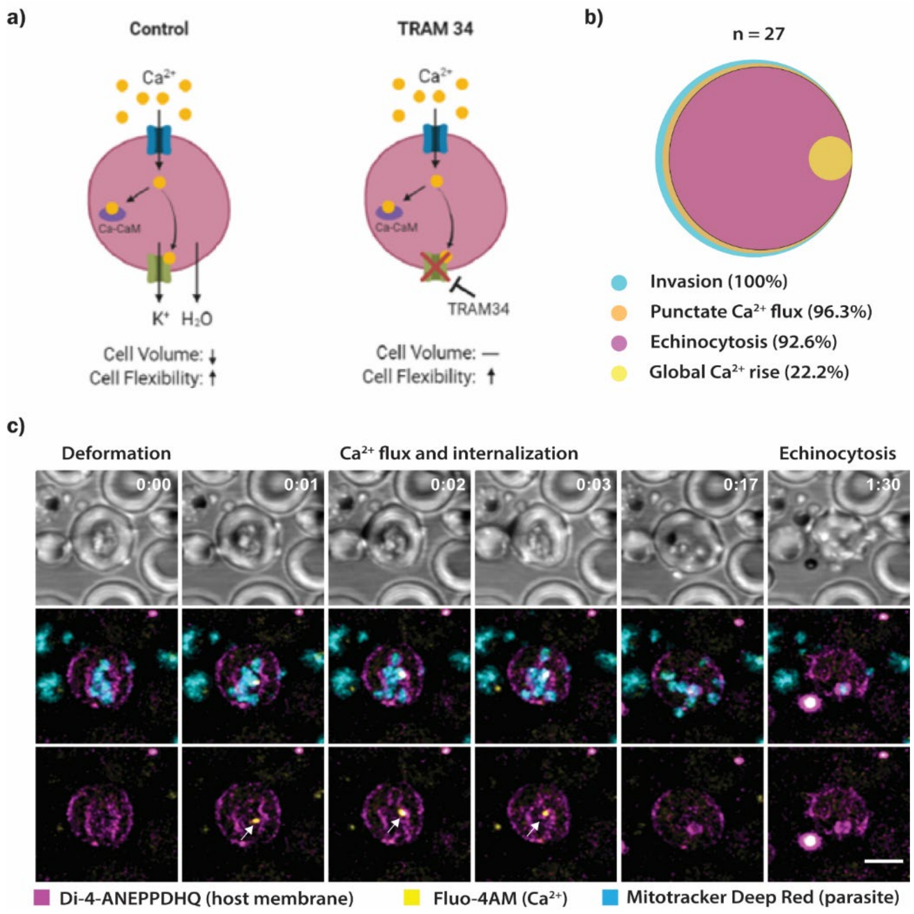

Supplementary Figure 4. Gardos channel activation via Ca^{2+} influx is not the cause for post-invasion echinocytosis. a) A schematic showing the effects of Ca^{2+} influx in the activation of calmodulin and Gardos channel, resulting in increased cell flexibility and decreased cell volume due to K^+ efflux and water loss. TRAM34 inhibits Gardos channel and prevents cell shrinkage due to water loss. b) A diagram representing invasion events in the presence of TRAM34. Echinocytosis still occurred in 92.6% of the invasion events (n = 27 from two independent experiments) following the punctate Ca^{2+} flux. Most of the echinocytosis events (70.4%) were not preceded by a global Ca^{2+} rise in the host cell. c) Snapshots from confocal microscopy showing echinocytosis after an invasion event in the presence of TRAM34. Scale bar: 5 µm.

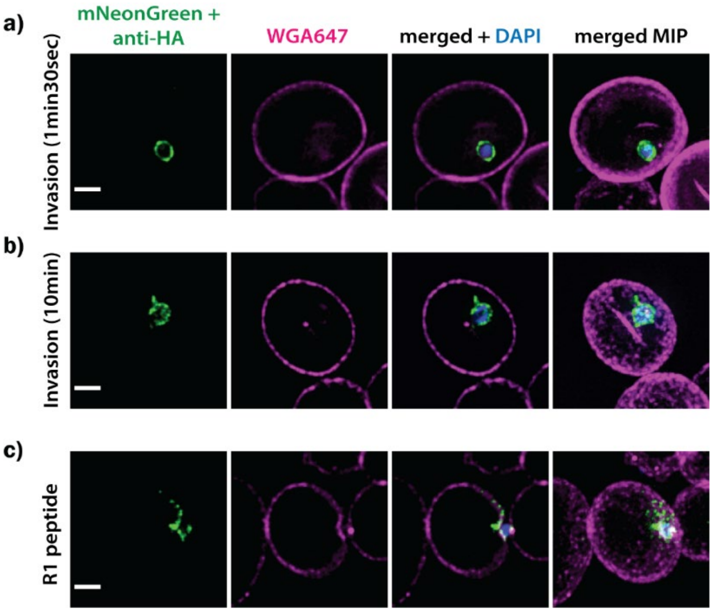

Supplementary Figure 5. RON3 becomes associated with the newly formed PVM upon invasion and is released onto surrounding erythrocyte membrane when tight junction formation is blocked. Representative snapshots from immunofluorescence assay displayed as single and merged channels, in slice view and maximum intensity projection (MIP). RON3 localization was marked by mNeonGreen enhanced with anti-HA (green), parasite nucleus by DAPI (blue), and red blood cell membrane by Wheat Germ Agglutinin 647 (magenta). Parasite-erythrocyte interactions were fixed at **a)** 1 min 30 sec, **b)** 10 min, or **c)** 1 min 30 sec in the presence of R1 peptide, after mixing of free merozoites and fresh red blood cells. Scale bars: 2 µm. The assay was first performed without membrane marker and then repeated with membrane marker.

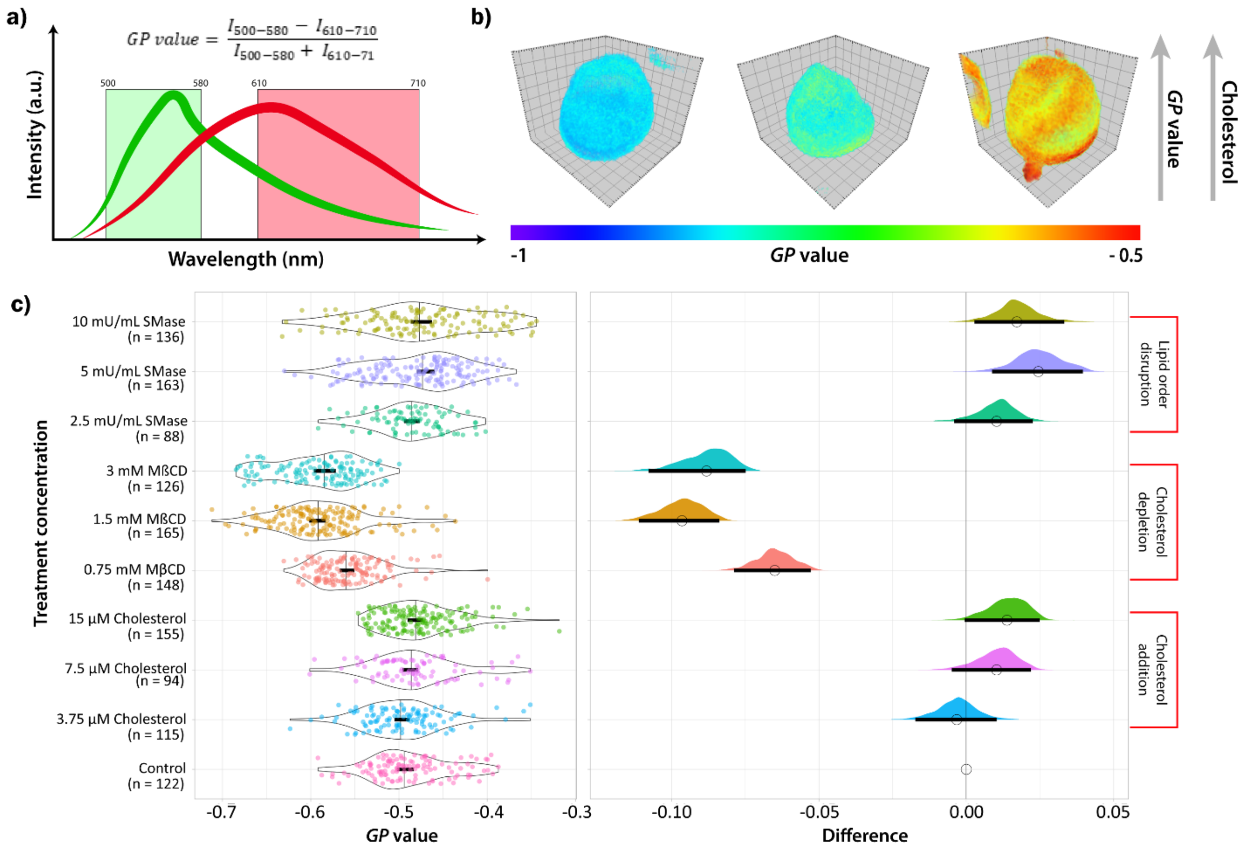

$$GP\ value = \frac{I_{500-580} - I_{610-710}}{I_{500-580} + I_{610-71}}$$

Supplementary Figure 6. Validation for Di-4-ANEPPDHQ as a cholesterol reporter. a-b) Fluorescence microscopy with simultaneous two-channels detection on erythrocytes labelled with Di-4-ANEPPDHQ reports on the relative membrane cholesterol content indicated by the calculated generalized polarization (*GP*) value. **c)** The erythrocytes were treated with different drugs to interrogate the effect of lipid order disruption, cholesterol depletion, and cholesterol addition to the average *GP* value. The *GP* values are significantly different on the cholesterol depleted cells, but not on the cells with disrupted ordered lipid regions and cholesterol addition. The results indicate that lipid order disruption does not affect the cholesterol content of the cells and that the erythrocytes are either already saturated with membrane cholesterol or the drug concentrations are not high enough to cause considerable increase in the cholesterol content.

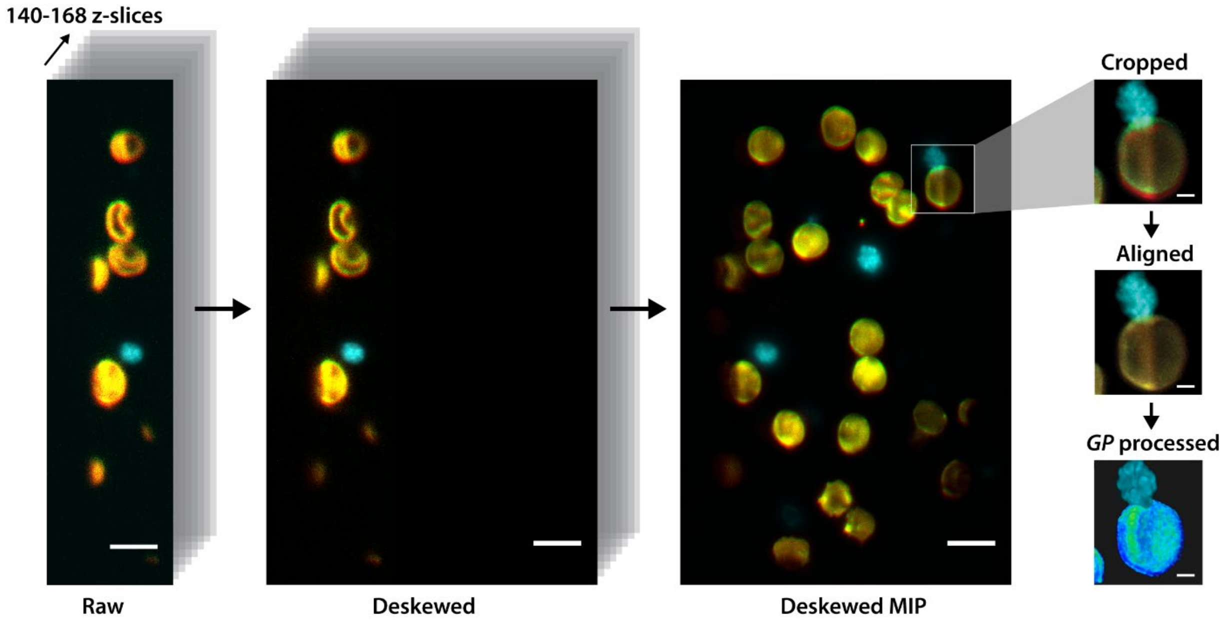

Supplementary Figure 7. Lattice light-sheet image acquisition and data processing steps for *GP* analysis. The image detection plane of the lattice light sheet microscopy is angled relative to the sample plane and the data were saved as an individual z-stack for each time-point. To process the data, each slice was deskewed and a maximum intensity projection (MIP) was generated to give an overview of the localization and condition of all the cells in the field of view. A region of interest was cropped and the channel acquired with camera B was aligned to the channel acquired with camera A. The aligned data were processed with a custom-written ImageJ/FIJI macro that transform the pixel value from intensity to generalized polarization (*GP*). Scale bars for the full field of view images and for the cropped images are 10 µm and 2 µm, respectively.

Supplementary Tables:

Supplementary Table 1. Parasite growth inhibition assay on MβCD treated erythrocytes

MβCD concentration (mM)	Average growth relative to control (from triplicate)		
	Experiment 1	Experiment 2	Experiment 3
0	100%	100%	100%
0.75	113%	88%	87%
1.5	57%	41%	21%
3	29%	18%	4%

Supplementary Table 2. Primer sequences used for construction of *P. falciparum* line expressing mNeonGreen-tagged RON3

Primer	Sequence
R3F1	5’-AGCTGCGGCCGCAAAATATAACTAAACCATCAGATC-3’
R3F2	5’-TATTGTATCTGGACTAACCATGG-3’
R3F3	5’-AGCTCTGCAGCCAAAAGCAGATATTATATCTTTATATAAAATTGTGG-3’
R3F4	5’-AGCTACTAGTATTCTTCTAACGTCAATACTGG-3’
