# Supplementary material for: 4D analysis of malaria parasite invasion offers insights into erythrocyte membrane remodeling and parasitophorous vacuole formation
Source: Nat Commun. 2021 Jun 15;12:3620. doi: 10.1038/s41467-021-23626-7 (PMC8206130; doi:10.1038/s41467-021-23626-7)
Supplement: Supplementary file 3 — Description of Additional Supplementary Information [file 41467_2021_23626_MOESM3_ESM.pdf]

Description of Additional Supplementary Files

File Name: Supplementary Movie 1

Description: Lattice light-sheet microscopy of P.falciparum invasion of erythrocytes. Accompaniment for Fig 1a.

File Name: Supplementary Movie 2

Description: Merozoite spinning following PVM sealing as imaged by lattice light-sheet microscopy. Accompaniment for Fig 1f-g.

File Name: Supplementary Movie 3

Description: Surface area and reduced volume measurements of host erythrocyte membrane as imaged by lattice lightsheet microscopy. Accompaniment for Fig 2c-d.

File Name: Supplementary Movie 4

Description: Lattice light-sheet microscopy data showing that calcium flux is apically located to the merozoite and occurs during membrane recoil. Accompaniment for Fig 3a and supplementary fig 2a-b.

File Name: Supplementary Movie 5

Description: Invasion inhibition using R1 peptide and cytochalasin D as imaged by lattice light-sheet microscopy. Accompaniment for Fig 3b-c.

File Name: Supplementary Movie 6

Description: Gaussian curvature of host erythrocyte membrane during invasion and R1 peptide inhibition as imaged by lattice light-sheet microscopy. Accompaniment for Fig 3e

File Name: Supplementary Movie 7

Description: Membrane disruption in the form of tethers and protrusions when invasion is inhibited

using R1 peptide or cytochalasin D as imaged by lattice light-sheet microscopy. Accompaniment for Fig 4c-d.

File Name: Supplementary Movie 8

Description: Imaging of RON3 distribution during invasion and R1 peptide inhibition as imaged by lattice light-sheet microscopy. Accompaniment for Fig 4f-g

File Name: Supplementary Movie 9

Description: Imaging of membrane cholesterol distribution during invasion as imaged by lattice light-sheet microscopy. Accompaniment for Fig 5a.

File Name: Supplementary Movie 10

Description: Imaging of parasite invasion and subsequent ejection of cholesterol depleted erythrocytes as imaged by lattice light-sheet microscopy. Accompaniment for Fig 5e.
