# Supplementary material for: 4D analysis of malaria parasite invasion offers insights into erythrocyte membrane remodeling and parasitophorous vacuole formation
Source: Nat Commun. 2021 Jun 15;12:3620. doi: 10.1038/s41467-021-23626-7 (PMC8206130; doi:10.1038/s41467-021-23626-7)
Supplement: Supplementary file 14 — Reporting Summary [file 41467_2021_23626_MOESM14_ESM.pdf]

nature research

Corresponding author(s): Kelly Rogers
Last updated by author(s): April 9, 2021

Reporting Summary

Nature Research wishes to improve the reproducibility of the work that we publish. This form provides structure for consistency and transparency in reporting. For further information on Nature Research policies, see our Editorial Policies and the Editorial Policy Checklist.

Statistics

For all statistical analyses, confirm that the following items are present in the figure legend, table legend, main text, or Methods section.

n/a	Confirmed	
☐	☒	The exact sample size (n) for each experimental group/condition, given as a discrete number and unit of measurement
☐	☒	A statement on whether measurements were taken from distinct samples or whether the same sample was measured repeatedly
☐	☒	The statistical test(s) used AND whether they are one- or two-sided<br>*Only common tests should be described solely by name; describe more complex techniques in the Methods section.*
☐	☒	A description of all covariates tested
☐	☒	A description of any assumptions or corrections, such as tests of normality and adjustment for multiple comparisons
☐	☒	A full description of the statistical parameters including central tendency (e.g. means) or other basic estimates (e.g. regression coefficient) AND variation (e.g. standard deviation) or associated estimates of uncertainty (e.g. confidence intervals)
☐	☒	For null hypothesis testing, the test statistic (e.g. F, t, r) with confidence intervals, effect sizes, degrees of freedom and P value noted<br>*Give P values as exact values whenever suitable.*
☒	☐	For Bayesian analysis, information on the choice of priors and Markov chain Monte Carlo settings
☒	☐	For hierarchical and complex designs, identification of the appropriate level for tests and full reporting of outcomes
☒	☐	Estimates of effect sizes (e.g. Cohen's d, Pearson's r), indicating how they were calculated

Our web collection on statistics for biologists contains articles on many of the points above.

Software and code

Policy information about availability of computer code

Data collection	The equipment and methods used to acquire data are fully disclosed in the manuscript. If required, more information (e.g. model numbers of equipment) can also be provided
Data analysis	Figures 1A,F; 3E; 4E-G were processed using ImageJ/FIJI. Figures 1A,B,E; 5A,E were processed and displayed as 3D volume rendered images with a clipping plane using IMARIS x64 v9.6.0 (Oxford Instruments); Figures 3A-C; 4C-D were dispalyed as an extended section using IMARIS x64 v9.6.0 (Oxford Instruments); Figure 3E was reconstructed with softWoRx 7.0 (GE Healthcare).The softwares used to analyze data were Excel (Microsoft 365 Apps for enterprise), Prism v.8.2 (GraphPad), ImageJ 1.52p (NIH), IMARIS v8 (Bitplane) and v9 (Oxford Instruments), Python 3.6 (Packages - numpy, matplotlib, pandas, seaborn), MATLAB 2019b (Mathworks), and PlotsOfDifferences (github/JoachimGoedhart). The methods used to analyze data are fully disclosed in the manuscript. If required, more information can also be provided. All data and customised Image J macros used by study are available on request from the authors.

For manuscripts utilizing custom algorithms or software that are central to the research but not yet described in published literature, software must be made available to editors and reviewers. We strongly encourage code deposition in a community repository (e.g. GitHub). See the Nature Research guidelines for submitting code & software for further information.

Data

Policy information about availability of data

All manuscripts must include a data availability statement. This statement should provide the following information, where applicable:

- Accession codes, unique identifiers, or web links for publicly available datasets
- A list of figures that have associated raw data
- A description of any restrictions on data availability

The raw data for all plots has been submitted as a supplementary file, names raw_data.xslx. The datasets generated during and/or analysed during the current study

are available from the corresponding author on reasonable request. All code used for data analysis, custom ImageJ macros and MATLAB code, are available upon request.

Field-specific reporting

Please select the one below that is the best fit for your research. If you are not sure, read the appropriate sections before making your selection.

- [x] Life sciences
- [ ] Behavioural & social sciences
- [ ] Ecological, evolutionary & environmental sciences

For a reference copy of the document with all sections, see nature.com/documents/nr-reporting-summary-flat.pdf

Life sciences study design

All studies must disclose on these points even when the disclosure is negative.

Sample size	Sample size calculations were not performed. Sample sizes for each experiment were chosen to be consistent with the field norms. Most of the results represent at least three independent experiments. In the case where the experiment was not repeated at least three times, there were either consistent observations across high number of individual events collected from the experiments (e.g. Figure 3C and Figure 4E), or the observations were complemented with other form of experiment to confirm the results (e.g. Figure 4F-G supported by Supplementary Figure 5).
Data exclusions	No data were excluded from the experiments in this study.
Replication	Wherever possible, experimental findings were replicated both through technical replicates and independent biological replicates. Figure 1A - n = 9 independent experiments; Figure 1C - n = 12 invasions, from 4 independent experiments; Figure 2D - n = 16 from 4 independent experiments; Figure 3D - Untreated - (n = 12 invasions from 3 independent experiments); Figure 3D - R1 Peptide - (n = 9 invasions from 3 independent experiments); Figure 3D - Cytochalasin D (n = 6 from 2 indepedent experiments); Figure 4A, Untreated (n = 14); MBCD (n = 13 invasion from 4 independent experiments); R1 Peptide (n = 19); Cytochalasin D (n = 14); Figure 4B, Untreated (n = 11); MBCD (n = 10); R1 Peptide (n = 20); Cytochalasin D (n = 23); Figure 5B - n = 8 invasions from 5 independent experiments. Further details can be provided on request.
Randomization	Randomization was not performed for experiments involving the effect of treatments on parasite invasion of red blood cells (eg. R1 peptide, MBCD or Cytochalasin D) as we assumed this would not alter data variance. As the experiments required the measurement of a very specific event, invasion, all invasion events were included in all measurements and, as such, not requiring any randomization.
Blinding	Blinding was not performed for the quantitation of parasite associated membrane time plots at Figures 1C, 3D and 5B, as these analyses used semi-automated macros performed in IMARIS. Blinding was not performed for any experiments involving the treatment of blood cells with different inhibitors/ treatments (R1 peptide, Cytochalasin D, MBCD) as we assumed that blinding the treatment of the red blood cells prior to invasion assays would not alter data variance. All analysis was performed using automated, or semi-automated, methods minimizing any biases that may arise. All thresholds and analysis metrics were preserved across all measurements.

Reporting for specific materials, systems and methods

We require information from authors about some types of materials, experimental systems and methods used in many studies. Here, indicate whether each material, system or method listed is relevant to your study. If you are not sure if a list item applies to your research, read the appropriate section before selecting a response.

Materials & experimental systems

n/a	Involved in the study	
[ ]	[x]	Antibodies
[ ]	[x]	Eukaryotic cell lines
[x]	[ ]	Palaeontology and archaeology
[x]	[ ]	Animals and other organisms
[x]	[ ]	Human research participants
[x]	[ ]	Clinical data
[x]	[ ]	Dual use research of concern

Methods

n/a	Involved in the study	
[x]	[ ]	ChIP-seq
[x]	[ ]	Flow cytometry
[x]	[ ]	MRI-based neuroimaging

Antibodies

Antibodies used	- Rat anti-HA primary antibody (Roche: Clone number 3F10)<br>- Donkey anti-rat IgG (H+L) secondary antibody conjugated to Alexa Fluor 488 (Invitrogen)
Validation	-Rat anti-HA 3F10 primary antibody from Roche was validated by the supplier through western blot.

Eukaryotic cell lines

Policy information about cell lines

Cell line source(s)	All cell lines are derived from P. falciparum 3D7, which is a commonly used lab adapted parasite strain. The parasite line was obtained from Dr. David Walliker Edinburgh University.
Authentication	The 3D7 line has been recently authenticated by whole genome sequencing.
Mycoplasma contamination	Cell lines were not tested for mycoplasma contamination but have been verified recently through whole genome sequencing.
Commonly misidentified lines (See ICLAC register)	3D7 is a routinely used P. falciparum lab strain across multiple laboratories and it is not listed in ICLAC.
